# Distinction between *Borrelia* and *Borreliella* is more robustly supported by molecular and phenotypic characteristics than all other neighbouring prokaryotic genera: Response to Margos' et al. "The genus *Borrelia* reloaded" (PLoS ONE 13(12): e0208432)

**DOI:** 10.1371/journal.pone.0221397

**Published:** 2019-08-27

**Authors:** Radhey S. Gupta

**Affiliations:** Department of Biochemistry and Biomedical Sciences, McMaster University, Hamilton, Ontario, Canada; Umeå University, SWEDEN

## Abstract

In a recent publication in PLOS ONE, Gabriele Margos and colleagues have questioned the division of the genus *Borrelia* into two genera on the basis that the differences in percentage of conserved proteins (POCP) between these two groups is >50%, which an earlier study has suggested as the threshold for differentiating prokaryotic genera. However, the POCP threshold is a poorly characterized and rarely used criterion for establishing distinction among prokaryotic genera. Detailed evaluation of the intergeneric POCP values for 37 genera from 3 different families (viz. *Enterobacteriaceae*- 24 genera, *Morganellaceae*-8 genera and *Cystobacteraceae*-5 genera) presented here shows that the POCP values for all genera within each of these families exceeded >58%. Thus, the suggested POCP threshold *is not a useful criterion for delimitation of genus boundary* and the objection by Margos *et al*. on this ground is invalid. Additionally, Margos *et al*. have questioned the specificities of ~15–20% of the conserved signature indels (CSIs) described in our work. However, as shown here, this concern is due to misunderstanding of the results and the CSIs in question are still highly-specific characteristics of the members of these genera and they provide important information regarding the evolutionary relationships of two new reptiles-echidna-related species viz. *Borrelia turcica* and Candidatus Borrelia tachyglossi to other *Borrelia* species. Results presented here show that both these species are deeper-branching members of the genus *Borrelia* and their placement within this genus is strongly supported by phylogenetic analyses and multiple uniquely shared CSIs with the other *Borrelia* species. Based on the large body of evidence derived from phylogenetic, genomic, molecular, phenotypic and clinical features, it is contended that the characteristics clearly distinguishing the *Borrelia* and *Borreliella* genera are far more numerous and of different kinds than those discerning most (all) other neighbouring genera of prokaryotes. Thus, the placement of these two groups of microorganisms into distinct genera, *Borrelia* and *Borreliella*, which clearly recognizes the differences among them, is highly appropriate and it should lead to a better understanding of the clinical, molecular and biological differences between these two important groups of microbes.

## Introduction

The family *Borreliaceae* includes species that are causative agents of Lyme disease (LD) and others that are causative agents of tick- and louse-borne relapsing fever (RF) [1–5]. Our earlier comprehensive phylogenomics and comparative studies on protein sequences from *Borreliaceae* genomes provided compelling evidence for the existence of two genetically distinct groups of organisms within this family [6,7]. Of these two groups, one group included all species that are causative agents of the clinically distinctive disorder known as RF, whereas the second group encompassed causative agents of LD along with some other closely related species [6]. The existence of these two groups was supported by different independent lines of evidence which included: (i) Distinct branching of the LD and RF groups of species in the 16S rRNA trees and multiple genome scale trees based on protein sequences [6–8]; (ii) Clear distinction of the LD and RF groups of species based on pairwise comparison of either the average nucleotide identity (ANI) or the average amino acid identity (AAI) of different genes/proteins from the *Borreliaceae* genomes [6,7]; (iii) Identification of >70 highly-specific molecular signatures consisting of conserved signature insertions/deletions (indels) (CSIs) in protein sequences and conserved signature proteins (CSPs) that are exclusively shared by different members of either the LD or the RF group of species [6,7]; and (iv) Several phenotypic characteristics known from earlier work including the distinct pathogenicity profiles of the two groups of organisms and differences in arthropod vectors used by them [1,2,4,5]. Based on the robust evidence provided by all of these analyses, we have previously proposed a division of the family *Borreliaceae* (and the genus *Borrelia*) into two main genera, *Borrelia* and *Borreliella* [6]. In this proposal, all of the species that are part of the RF group were retained within the genus *Borrelia*, whereas all species related to the LD group were placed into a new genus called *Borreliella* [6]. This latter group of species is widely referred to by the name "*Borrelia burgdorferi sensu lato*", recognizing their distinctness from the RF group of species [1,2,5,9].

Recently, Margos *et al*. [10,11] have analyzed the genome sequences from two new *Borreliaceae* species, viz. *Borrelia turcica* and Candidatus Borrelia tachyglossi, which are associated with reptiles and echidna. In their publication, Margos *et al*. [10] acknowledge that the LD and RF groups of species “have different clinical, biological, and epidemiological characteristics, and phylogenetic data is concordant with this, demonstrating that these two groups are genetically similar yet distinct and form independent monophyletic sister clades that once shared a common ancestor”. Additionally, they state that the proposal by Adeolu and Gupta [6] to divide the genus *Borrelia* into two genera “was largely based on the identification of conserved signature insertions/deletions (indels) (CSIs) and conserved signature proteins (CSPs) that are differentially present in the LD or RF *Borrelia* genogroup, as well as average nucleotide identity (ANI) values calculated between whole genomes of 18 *Borrelia* species including eight LD species and ten RF species … it is uncontested that these differences exist between LD and RF *Borrelia*”.

Margos *et al*. [10] have questioned the division of genus *Borrelia* into two genera on three accounts. Their main argument for questioning the division is based on the consideration that the differences in the ANI or AAI values between these two groups, shown in our work as part of the evidence indicating that these two groups of species differ from each other [6,7], are not suitable means for differentiation of prokaryotic genera. Instead, they assert that a method proposed by Qin *et al*. [12] based on percentage of conserved proteins (POCP) between genomes from different species is a more reliable means for the determination of a genus level boundary. In addition to this main objection, the authors also criticize our work on two other grounds: (i) that the methodology used in our work only identifies CSIs and CSPs that are exclusive to only one *Borrelia* genogroup and it precludes the detection of those characteristics that are shared non-exclusively between both genogroups, and (ii) that upon inclusion of sequence information for the two new *Borrelia* species, about 17–20% of the previously reported 53 CSIs are unable to differentiate between the LD and the RF groups of species. I discuss below our responses to all of these criticisms and specifically the problem of using or relying on the suggested POCP threshold as a criterion for the delimitation of prokaryotic genera, which is the main basis of Margos *et al*.’s [10] resistance to our division of the genus *Borrelia* into two genera.

## Materials and methods

Construction of the phylogenetic tree based on the core genome proteins and the calculation of percentage of conserved proteins (POCP) between different genomes was carried out using an internally developed software pipeline described in earlier work [13–15]. Information regarding genome sequences for different species from the families *Enterobacteriaceae* [15], *Morganellaceae* [14] and *Cystobacteraceae*, for which the POCP values were calculated is provided in S1 and S2 Tables. Briefly, using the CD-HIT program [16], proteins sharing a minimum of 50% sequence identity and sequence length were identified in different genomes. Based on this information, the POCP between different pairs of genomes was calculated as described by Qin *et al*. [12]. Multiple sequence alignment (MSA) of the proteins which were found in at least 80% of the input genomes (a total of 703 protein families) were created using Clustal Omega [17]. For phylogenetic analysis, the sequence alignments were trimmed using TrimAl [18] before their concatenation into a single file. The combined sequence for the 703 core genome proteins, which after trimming consisted of 248452 aligned amino acids, was utilized for phylogenetic analysis. A maximum likelihood (ML) tree based on this sequence alignment was constructed and optimized in RAxML 8 as described in our earlier work [13–15].

The 16S rRNA gene sequences for different *Borreliaceae* species were downloaded from All-Species Living Tree Project [19] and aligned using ClustalX2. The tree was constructed using the Maximum-likelihood (ML) method in MEGA6 [20]. Updating of the sequence information and group specificity of different CSIs and CSPs was carried out by performing BLASTp searches on the sequences of the indicated proteins. Formatting of the sequence alignment files was carried out using SIG_CREATE and SIG_STYLE programs described in our work [21]. It should be mentioned that based on different lines of evidence, the following *Borrelia* species (viz. *B*. *bissettii*, *B*. *lanei*, *B*. *mayonii* and *B*. *yangtzensis*) consistently group with the LD group. Unlike the other LD group of species, which are now transferred to the genus *Borreliella* [6], the proposal to reclassify these four species to the genus Borreliella has not yet been made. However, in the interim, to avoid any confusion due to the grouping of these *Borrelia* species within other *Borreliella* species, the genus name of these species is abbreviated as “Bor.” in the manuscript and different Figs.

## Results and discussion

### The inadequacy of using a 50% POCP threshold for genus level boundaries

Prokaryotic systematics involves assemblage of organisms into groups of different ranks from most inclusive to least inclusive (e.g. Phylum, Class, Order, Family, Genus and Species) on the basis of their observed similarities and differences and phylogenetic/evolutionary relationships [22–25]. Species are the basic unit of any biological classification scheme. For prokaryotic organisms, although a formal definition of “the species” is lacking, for practical purposes, it is now generally accepted that strains showing >70% similarity in DNA-DNA hybridization values, or >98.65% sequence similarity in 16S rRNA, or those exhibiting >95% similarity in ANI values provide comparable means for delimiting a prokaryotic species and for identification of new species [23,25–33]. In contrast to these accepted criteria for species delimitation, *there are no commonly accepted or used criteria for identification of genus or higher level taxa* [34]. A genus is commonly defined as *“a monophyletic grouping of species with many characters in common”*[22,35]. Further, there is a general consensus that the division into higher taxonomic ranks including genus level taxon should reflect phylogenetic relationships.

While there are no accepted criteria for genus level boundaries, some authors have suggested that the 16S rRNA similarity values between 94.5% and 86.6% [26] or the POCP values <50% [12] can be used as thresholds for differentiation among genera. However, these suggestions are based on studies using a limited number of prokaryotic taxa and the general utilities of these methods (or suggested thresholds) for delimitation of prokaryotic genera remains to be properly evaluated. Let us now specifically consider the utility of using the 50% POCP threshold value as a genus level boundary, which Margos *et al*. [10] contend provides a more suitable method for demarcation of prokaryotic genera. The study by Qin *et al*. [12], which suggested the use of POCP values for genus level delimitation was based on a limited number of prokaryotic taxa and the inferences based on it suffer from a number of drawbacks: (i) Interspecies POCP comparison in this study was carried out for only 17 genera. Of these, several genera such as *Bacillus*, *Lactobacillus* and *Clostridium* are highly polyphyletic and only a selected group of closely related species were chosen from them to represent the entire genera [12]. Due to arbitrary delimitation of these genera to a small group of selected species, the closest relatives of these genera, which are other species from the same genera, were not considered in either the interspecies or intergeneric POCP comparisons. (ii) Intergeneric POCP comparisons were carried out with only 1 arbitrarily chosen species from these 17 genera to only single species from other genera, families and orders of bacteria [12]. As many of these latter comparisons were made for species that are part of different families or orders of bacteria, the POCP values obtained for them do not reflect intergeneric differences, but rather family or order level differences. The latter values are expected to be lower than intergeneric differences and the results from such comparisons should not have been included in the comparison of intergeneric POCP values as they artificially lower the observed intergeneric values. (iii) Several genera used for interspecies comparison viz. *Thermotogae*, *Clostridium*, *Mycobacterium*, for which the POCP values were indicated to be higher than 50%, have since been divided into multiple genera [13,36–38] indicating that the POCP threshold is not a useful or required criterion for genus level separation.

**Fig 1:**
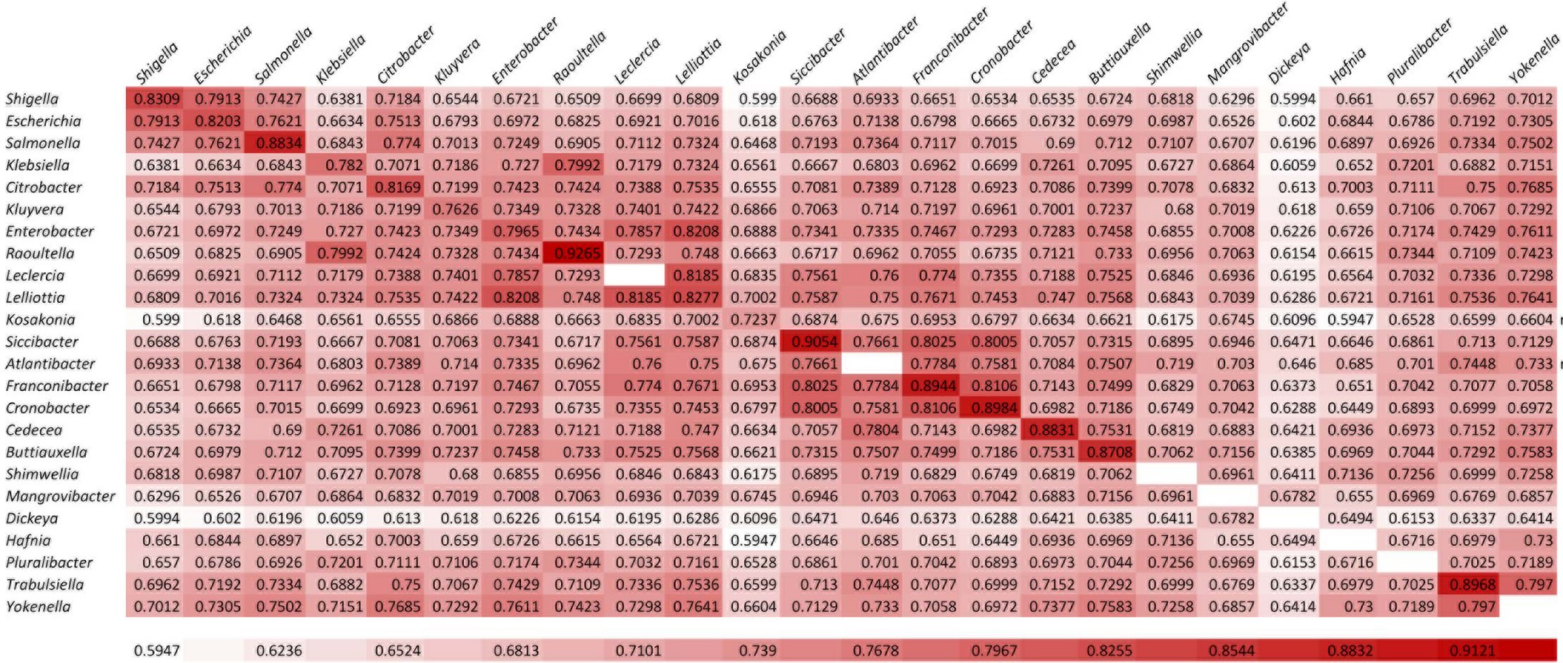
A comparison matrix showing the averages of the percentage of conserved proteins (POCP) within and between different genera of the family *Enterobacteriaceae*. POCP was determined for all genome sequenced species from the family *Enterobacteriaceae* detailed in our earlier work [26]. The values along the diagonal shows the average POCP values for different species within a given genus (i.e. interspecies values), whereas all other values represent average intergeneric POCP values for different genera within this family. The blank cells indicate that only a single species was available for these genera and hence their interspecies values could not be calculated.

To further evaluate the usefulness of POCP values for genus level separation/boundary, we have independently determined interspecies and intergeneric POCP values for a number of families each containing multiple genera. Three well-studied families that we have examined in this regard include the family *Enterobacteriaceae* containing 24 genera [15], the family *Morganellaceae* containing 8 genera [14], and the family *Cystobacteraceae* containing 5 genera [39]. For all of these families, pairwise interspecies and intergeneric POCP values were determined for all species for which genome sequences were available. From the pairwise POCP matrix, average POCP values were determined for different species within each genus (interspecies POCP values) and for different genera within each of these three families (intergeneric POCP values). The results of these comparisons for the family *Enterobacteriaceae* are presented in the pairwise POCP matrix in Fig 1.

As seen from the matrix in Fig 1, the intergeneric POCP values for all 24 genera that are part of this family range from a low of 59.4% to a high of 82.0% and they are all higher than 50%. Similarly, the intergeneric POCP values for the 8 genera that are part of the family *Morganellaceae* range from 58.7% to 84.5% (S3 Table), and for the family *Cystobacteraceae*, they range from 61.9%– 82.4% (S3 Table). Thus, if a POCP cut-off value of <50% was to be used for genus level boundary, then all of the different genera present within each of these three families would be part of a single genus. These results demonstrate that the usefulness of the 50% POCP threshold value for determination of genus level boundaries is very limited, if any.

Margos *et al*. [10] have also presented a comparison of the POCP values for the *Borrelia* and *Borreliella* genera along with some other genera within the phylum Spirochaeta. However, of the four other genera for which the POCP comparisons were made, *Brachyspira* and *Leptospira* are part of two separate orders viz. *Brachyspirales* and *Leptospirales* within the phylum Spirochaeta [40,41]. Based on the 16S rRNA sequence similarity comparisons, Yarza *et al*. [26] have previously noted that the species from these two orders, which are very distantly related to each other as well as other orders within the phylum Spirochaeta, should in fact be assigned *class* level ranks within the phylum. Thus, a comparison of the POCP values for these two genera with the other genera is misleading as they provide an indication of the order or class level differences and not intergeneric differences. The other two genera included in the comparison are *Treponema* and *Spirochaeta*. Although both of these genera are part of the family *Spirochaetaceae* [40,41], in phylogenetic trees, members of these genera form different clades indicating extensive divergence (unpublished results) [19,41,42]. Based on the results shown by Margos *et al*. (S1 Table of their publication) [10], the interspecies POCP values for members of these two genera are mostly in the range of 20–40% with an average POCP value of 33.7% for the *Treponema* species and 35.5% for the *Spirochaeta* species. Based on the 50% POCP threshold value for genus level boundaries, the species from both *Treponema* and *Spirochaeta* genera should each be divided into multiple genera. These results again point to the inadequacy of using the suggested POCP threshold value as a reliable means for the genus level boundaries.

**Fig 2:**
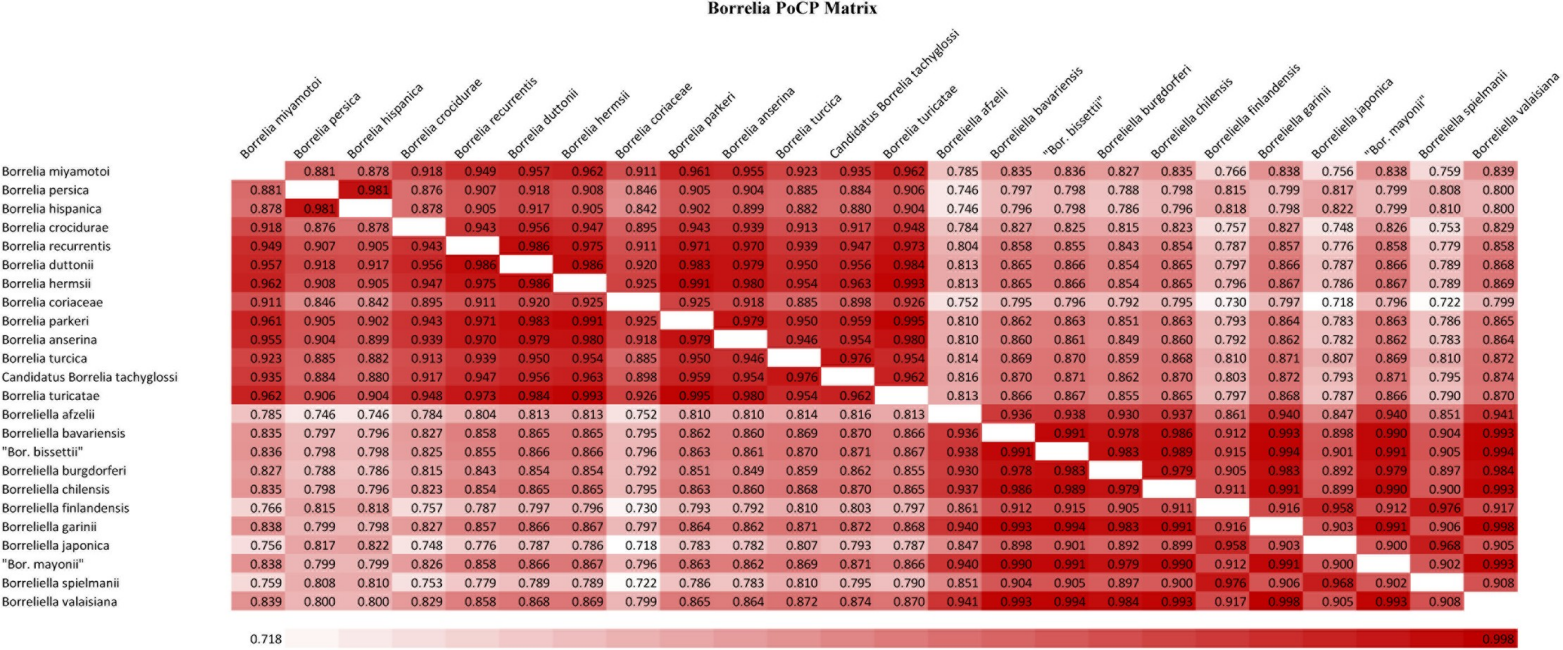
A pair-wise comparison matrix based on percentage of conserved proteins (POCP) in chromosomal genes from different genome sequenced *Borreliaceae* species. The matrix was constructed using an internally developed pipeline [13,14]. Genome pairs sharing higher POCP are shaded more darkly (red). Based on their POCP values, species belonging to the family *Borreliaceae* form two main groups, with one group containing all of the LD and related species (or *Borreliella*), and the other encompassing RF group of species together with the reptile-and echidna- associated species *B*. *turcica* and Candidatus Borrelia tachyglossi (genus *Borrelia*).

Although a specific POCP value is not very useful for establishing a genus level boundary, a comparison matrix based on POCP, similar to the matrices based on ANI or AAI values, can still provide an overall indication of the genomic similarity and differences between two closely related groups of species. In the POCP matrix presented by Margos *et al*. [10], while the species from the genus *Borreliella* (LD group) exhibited a high degree of similarity to each other, the species from the *Borrelia* (RF) group exhibited considerable variability and this group was not clearly differentiated. However, the POCP matrix constructed by Margos *et al*. [10] was based on genome sequences that included genes present on both the linear chromosomes as well as different plasmids. The distribution of plasmids is highly variable in different *Borreliaceae* species/strains unlike the conservation of linear chromosome structure and chromosomal genes, [5,43–46] and inclusion of plasmid sequences will introduce considerable variability in genome sequence or POCP comparison. Thus, in order to reliably compare the POCP values among different species, such comparisons should be based only on the chromosomal genes not including the plasmid genes. A POCP matrix for the *Borreliaceae* species based on genes present on chromosomal sequences is presented in Fig 2. As seen, this matrix clearly distinguishes the *Borreliaceae* species into two groups corresponding to the *Borrelia* and *Borreliella* genera. Based on this matrix, the average POCP for species from the genera *Borrelia* and *Borreliella* are 93.4% and 94.7%, respectively, whereas the average POCP value between these two groups is only 82.2%. Thus, a comparison of the POCP values based on chromosomal genes actually supports the genetic distinction between the *Borrelia* and *Borreliella* genera.

### Specificity of the molecular signatures for the *Borrelia* and *Borreliella* genera

In the Margos *et al*. [10] paper, concerns were also raised regarding our methodology for identifying CSIs and CSPs, which they assert only considered those molecular signatures which were exclusively found in one *Borrelia* genogroup and precluded detection of such characteristics that are shared non-exclusively between both genogroups. However, in our original work, in addition to the CSIs and CSPs that are specific for the two main groups (viz. *Borrelia* and *Borreliella*), we also reported 31 CSIs and 82 CSPs that are specifically found in all *Borreliaceae* species [6,41]. This information was also provided and emphasized in our rebuttal response [7] to an earlier criticism of our work by these authors [47]. By non-exclusive, however, if Margos *et al*. [10] mean that the CSIs or CSPs are commonly shared by only some members from each of the two main clades of *Borreliaceae* species, then in our work we have not come across significant number of such characteristics showing any specific pattern. However, isolated characteristics of this kind can result from lateral gene transfers and they are not useful for understanding evolutionary relationships or for taxonomic purposes [32,48].

**Fig 3:**
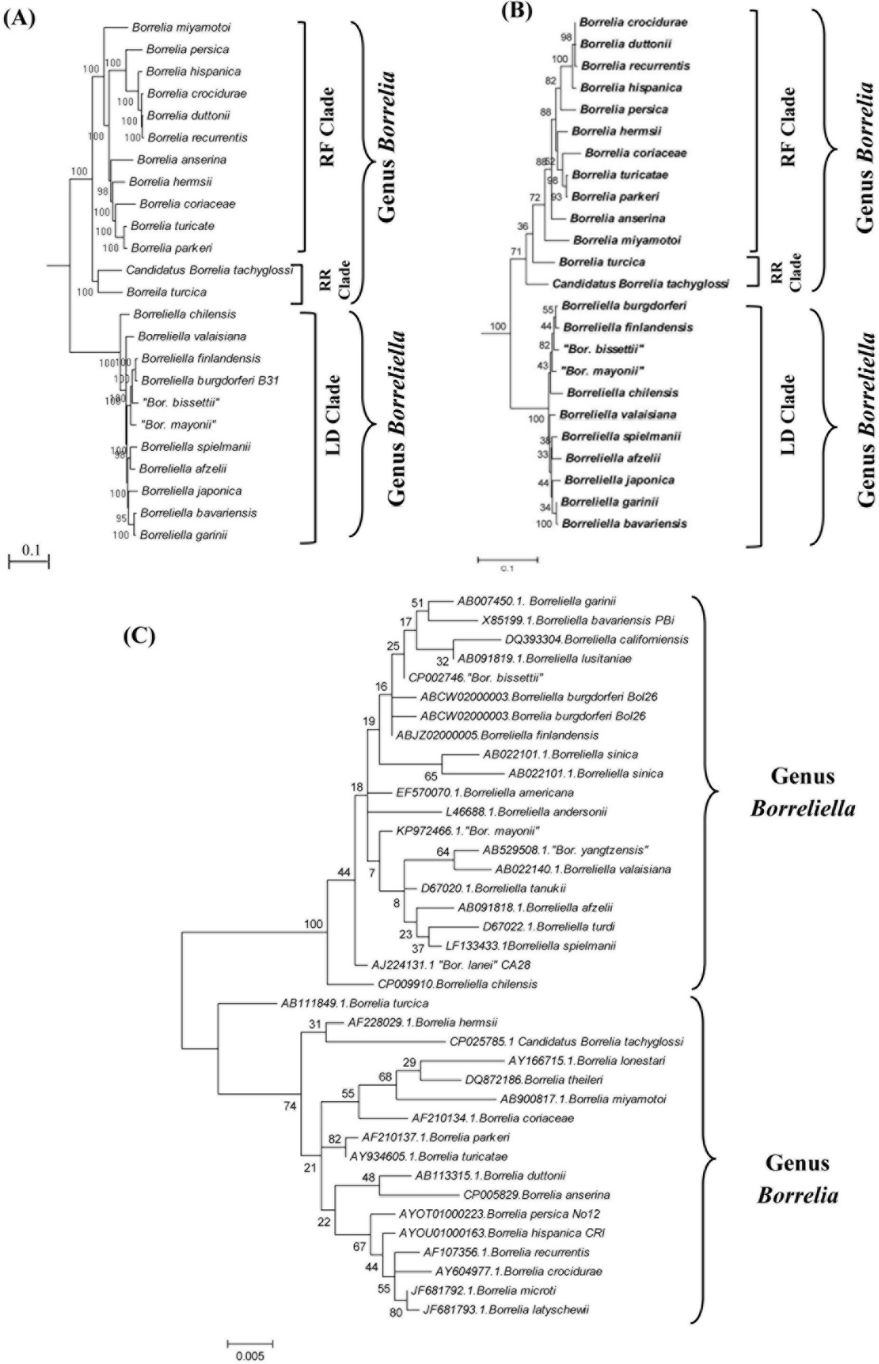
Phylogenetic trees showing the branching of *Borreliaceae* species. (A) A maximum-likelihood (ML) tree based on concatenated sequences of 703 core proteins found in the genomes of *Borreliaceae* species; (B) A tree based on sequence alignment for the RNA polymerase β’- subunit (RpoC protein). (C) A ML tree for *Borreliaceae* species based on 16S rRNA gene sequences.

Margos *et al*. [10] also state that between 17–20% of the CSIs identified by us are not specific for *Borrelia* or *Borreliella* genera and do not differentiate between these two groups. However, subsequent to our earlier work describing the specificities of the CSIs for two *Borreliaceae* genera [6], genome sequences have become available for two new *Borrelia* isolates viz. *B*. *turcica* and Candidatus Borrelia tachyglossi [10,11], and they were included by Margos *et al*. [10] in their analyses. Of these two species/strains, *B*. *turcica* is associated with reptiles whereas Candidatus Borrelia tachyglossi was isolated from an echidna (*Tachyglossus aculeatus*) species [11]. In phylogenetic trees based on 16S rRNA sequences as well as multiple genome-scale phylogenetic trees and trees based on individual protein sequences (Fig 3), these two species form deeper branching lineages of the *Borrelia* (RF) clade [10,11]. Although in a number of trees, particularly those based on large datasets of protein sequences (Fig 3A) [10], these two species form a clade, such an association is often not seen in trees based on sequences for many individual proteins (see Fig 3B) or in the tree based on 16S rRNA gene sequences (Fig 3C). However, we will refer to *B*. *turcica* and Candidatus Borrelia tachyglossi as the Reptiles-related (RR) group/clade in this work.

The inclusion of these two new species in the dataset, depending upon their branching position, is expected to alter the specificity of some of the identified signatures. In our earlier rebuttal response to Margos *et al*. [7,47], we had clearly outlined the different scenarios of how the inclusion of sequence information for the RR group of species, depending upon their branching positon within the family *Borreliaceae*, will affect the group-specificity of some of the identified CSIs. It was stated that if “the RR species/strains branch either within the RF group or as an outgroup of this clade, then such a group of species is expected to contain either some or all of the signatures for the RF clade, but generally none for the LD group”[7]. This is exactly what is observed upon the inclusion of sequence information for *B*. *turcica* and Candidatus Borrelia tachyglossi sequences. Thus, the questions raised by Margos *et al*. [10], regarding the specificities of some of the CSIs indicate that they are misinterpreting the results for the species distribution of the indicated CSIs.

**Fig 4:**
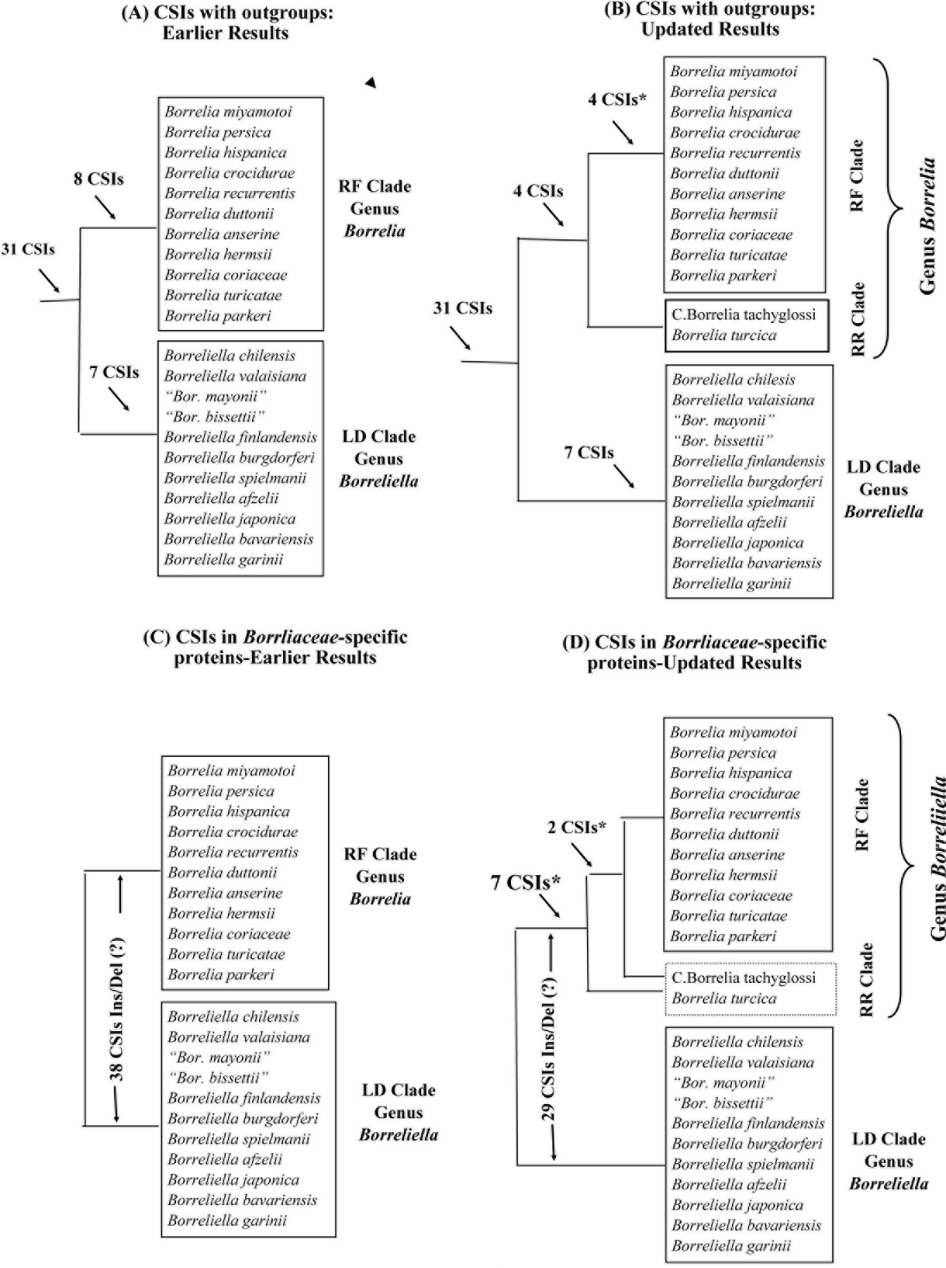
A summary diagram showing the species specificities of different CSIs reported in our earlier work [6]. The CSIs described in our earlier work were of two kinds. Panels (A) and (B) present the results for CSIs, where sequence information for outgroup species was available, whereas panels (C) and (D) show results for CSIs which are found in proteins that are limited to the *Borreliaceae* species (i.e. no homologs in any outgroup species). Panels (A) and (C) show the results as reported earlier [6], whereas panels (B) and (D) show how the observed specificities of the CSIs have been affected upon inclusion of sequences for *B*. *turcica* and Candidatus Borrelia tachyglossi. The asterisks (*) marks the CSIs whose specificities have been questioned by Margos *et al*. [10]. As shown here and as discussed in the text, these CSIs remain specific for the RF group (genus *Borrelia*) in addition to providing important information regarding the branching or phylogenetic placement of *B*. *turcica* and Candidatus Borrelia tachyglossi within the genus *Borrelia* and family *Borreliaceae*.

To go over their objections, let us consider the results for different CSIs that were reported previously and how they have been affected upon the inclusion of sequence information for *B*. *turcica* and Candidatus Borrelia tachyglossi. As noted earlier, 31 identified CSIs were specific for the family *Borreliaceae* (Table 2 in Ref. [6]). These CSIs, as expected, are also present in protein homologs from *B*. *turcica* and Candidatus Borrelia tachyglossi (results not shown). The remaining CSIs, which distinguished the two main groups within the family *Borreliaceae* were/are of two kinds. Of these, the first category of 15 CSIs are in proteins whose homologs besides the family *Borreliaceae* are also found in other bacteria (i.e. outgroup species) (Fig 4A and 4B). Based on the presence or absence of these CSIs in the outgroup species, one can infer whether these CSIs represent an insert(s) or deletion(s) and at what specific stage in the evolution of *Borreliaceae* family the genetic changes responsible for these CSIs have occurred [6,7,32,41]. Of these 15 CSIs, based on the available information, 7 CSIs were indicated to be specific for the LD group, whereas in the remaining 8, the genetic changes leading to the CSIs occurred in the lineage leading to the RF group of species. Upon inclusion of sequence information for *B*. *turcica* and Candidatus Borrelia tachyglossi, which form deeper branching lineages of the RF group, no changes were observed in the specificities of any of the CSIs specific for the LD group and the homologs of the two new species lacked these CSIs (see Table 1).

**Table 1:** Conserved signature indels (CSIs) found in widely distributed proteins that are specific for the members of the Lyme disease Borrelia (i.e. genus *Borreliella*).

Protein Name	GI Number	*B*.*burgdorferi* B31 locus	Indel Size	Indel Position
Recombinase A	492960118	BB_0131	1 aa ins	228–272
Trigger factor Tig	386854012	BB_0347	2 aa ins	106–142
Chemotaxis protein CheY	15594760	BB_0415	1 aa del	197–231
DNA polymerase III subunit beta	410679212	BB_0438	1 aa del	135–176
Translation factor Sua5	15595079	BB_0610	2 aa ins	149–182
Ferrous iron transporter A	51598605	BB_0730	1 aa del	88–126
Glucose-6-phosphate isomerase	493478887	BB_0734	1 aa ins	81–134

These 7 CSIs described in our earlier work [6] are uniquely shared by different genome-sequenced *Borreliella* (or LD group) species. Updating of sequence information for these CSIs show that they are not found in any species from the genus *Borrelia* (RF group) including *Borrelia turcica* and Candidatus Borrelia tachyglossi. Sequence alignments for these CSIs have been presented in earlier work [6].

**Fig 5:**
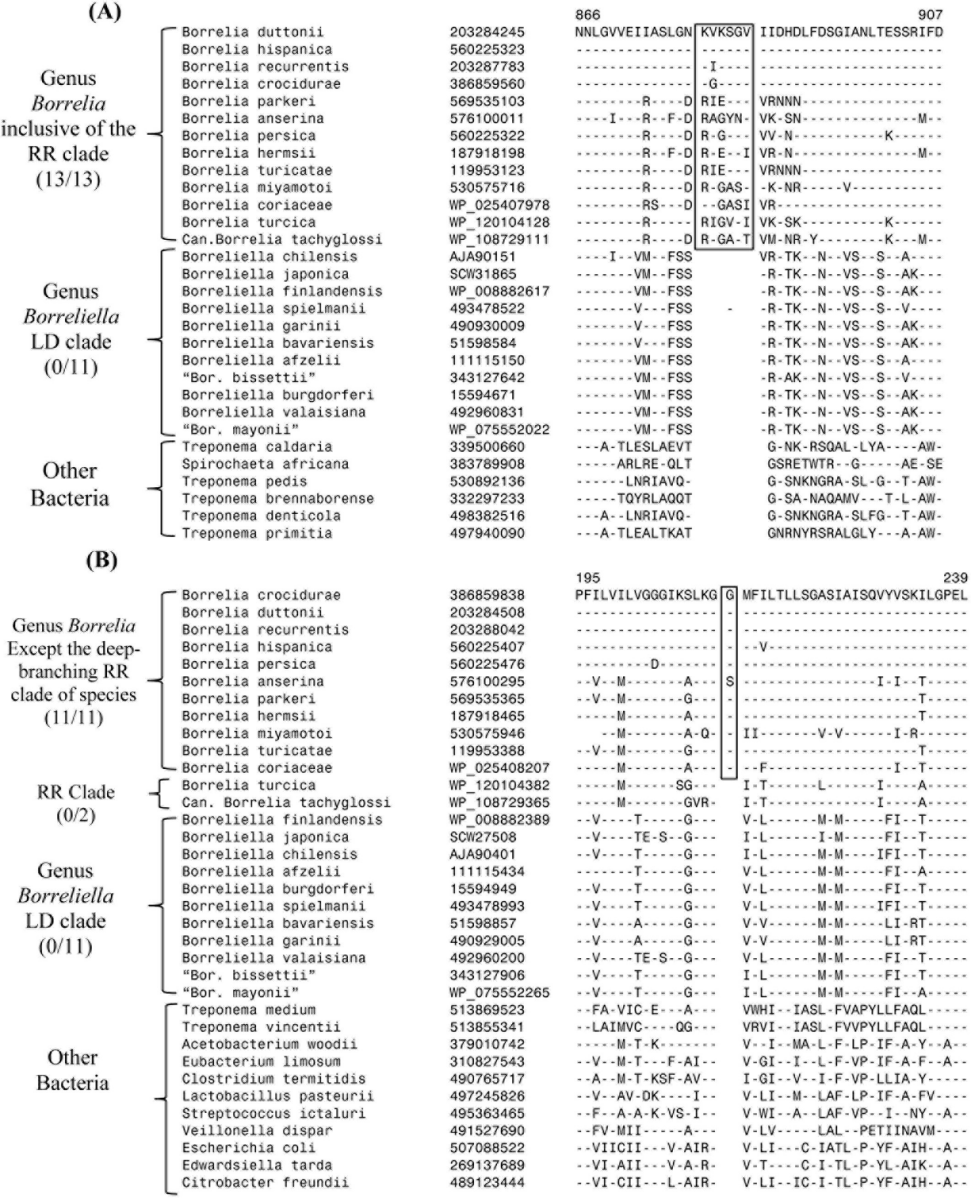
Partial sequence alignments of two CSIs in proteins with outgroup species that were previously reported as specific for the RF clade. Panel (A) shows a 6 aa insert in a hypothetical protein BDU327 (BB_0326) that is specifically found in all members of the genus *Borrelia* including *B*. *turcica* and Candidatus Borrelia tachyglossi. (B) This panel shows a 1 aa insert in the L-lactate permease protein, which is only shared by all RF clade species but is absent in the *B*. *turcica* and Candidatus Borrelia tachyglossi homologs, which are deeper branching members of the genus *Borrelia* (see Figs 3 and 4). Dashes (-) in all alignments shows sequence identity with the amino acids on the top line.

However, the CSIs which were previously indicated to be specific for the RF clade showed two patterns. Of these, 4 CSIs are commonly shared by all members of the RF group as well as *B*. *turcica* and Candidatus Borrelia tachyglossi (RR group), whereas the remaining 4 CSIs were only found in the RF group of species and not found in the two deeper branching RR group of species. In Fig 5, an example of CSIs showing the two types of patterns are presented. Information regarding the species specificities of all other CSIs for this group is presented in Table 2. The species distribution pattern of the CSIs for this group is exactly as we had predicted previously and the observed results, independent of the phylogenetic trees, strongly support the following inferences: (i) RR group of species, i.e. *B*. *turcica* and Candidatus Borrelia tachyglossi, are specifically associated with the RF group (i.e. genus *Borrelia*) as indicated by the 4 CSIs they uniquely share with the other RF group of species (Table 2A; Figs 4 and 5); (ii) *B*. *turcica* and Candidatus Borrelia tachyglossi are earlier branching members of the genus *Borrelia* and the genetic changes in the 4 CSIs that are absent in these two species have occurred in a common ancestor of the other *Borrelia* species, after the divergence of these two species (Table 2B; Figs 4 and 5). Thus, the species distribution patterns of the CSIs, upon inclusion of sequence information for *B*. *turcica* and Candidatus Borrelia tachyglossi, rather than showing any lack of specificity, provide important information clarifying and strongly supporting the observed evolutionary relationship of these species to the other *Borreliaceae* species (Fig 4). The CSIs whose specificities are questioned by Margos *et al*. [10] are marked by an asterisk (*) in Fig 4.

**Table 2:** Conserved signature indels in proteins that are specific for either all members of the Genus *Borrelia* or those lacking in the deeper branching *Borrelia turcica* and Candidatus Borrelia tachyglossi.

Protein Name	GI Number	Indel Size	*B*.*burgdorferi* B31 locus	Indel Position
**(A) CSIs specific for all members of the Genus *Borrelia***				
Hypothetical protein BRE16	203287484	3 aa ins	BB_0011	64–98
Hypothetical protein BDU327	203284245	6 aa ins	BB_0326	866–907
1-phosphofructokinase	203288064	1 aa del	BB_0630	101–139
GTP-binding protein	203288075	2 aa ins	BB_0643	42–87
**(B) CSIs specific for the Genus *Borrelia* except deeper branching *B*. *turcica* and Candidatus Borrelia tachyglossi**				
Nicotinamide-nucleotide adenylyltransferase	187918635	1 aa del	BB_0782	31–61
Hypothetical protein BT0471^1^	119953261	1 aa del	BB_0471	216–261
L-lactate permease	386859838	1 aa ins	BB_0604	195–239
Sodium/panthothenate symporter	119953591	1 aa ins	BB_0814	421–454

These 8 CSIs were described as specific for the RF clade of *Borrelia* species in our earlier work [6]. Upon inclusion of sequence information for including *Borrelia turcica* and Candidatus Borrelia tachyglossi, 4 of these CSIs are also uniquely shared by these two species, whereas the other four CSIs listed below are absent in these two deeper branching species. These CSIs provide evidence that both *Borrelia turcica* and Candidatus Borrelia tachyglossi are members of the genus *Borrelia* and constitute deeper branching lineages of this genus (see summary Fig 4). Sequence alignment of one CSIs’ of each kind is presented in Fig 5.

The remaining 38 CSIs, which constitute the second category, are present in proteins that are found only in different *Borreliaceae* species [6]. Although these CSIs differentiate members of the LD and the RF group of species, due to the absence of these proteins in outgroup species, it is difficult to determine whether the genetic changes giving rise of these CSIs represent insertion(s) in the LD (RF) group, or deletion(s) in the RF (LD) group (see Fig 4C). Thus, Margos *et al*. [10] are misinterpreting the results for these CSIs, when they indicate that a specific CSI of this kind is an insert or a deletion in the RF or the LD group of species. Nonetheless, with the inclusion of sequences for *B*. *turcica* and Candidatus Borrelia tachyglossi, which are deeper branching species associated with the RF group, depending upon where the genetic changes responsible for these CSIs have occurred, the species distribution pattern of some of these CSIs will be altered. The presence and absence of the indels in all 38 CSIs from this category and their correct interpretation is provided in Table 3.

**Table 3:** Conserved signature indels in *Borreliaceae*-specific proteins distinguishing *Borrelia* and *Borreliella* and showing deeper branching of the RR group of species within the genus *Borrelia*.

			*Borrelia*		*Borreliella*	
Protein Name	*B*.*B* 31 Locus	Indel Size	RF Clade	RR Species	LD Clade	Interpretation
Hypothetical protein	BB_0028	2 aa	-	-	+	Distinguishes two genera
Hypothetical protein	BB_0028	1 aa	-	-	+	Distinguishes two genera
Hypothetical protein BRE47	BB_0044	5 aa	-	-	+	Distinguishes two genera
L-proline transport system ATP-binding protein	BB_0146	1 aa	-	-	+	Distinguishes two genera
Penicillin-binding protein	BB_0136	1 aa	-	-	+	Distinguishes two genera
Hypothetical protein Q7M131	BB_0125	1 aa	+	+	-	Distinguishes two genera
Hypothetical protein BT0110	BB_0110	2 aa	-	-	+	Distinguishes two genera
Hypothetical protein BT0110	BB_0110	2 aa	-	N/A	+	Distinguishes two genera
Glutamate racemase	BB_0100	6 aa	-	-	+	Distinguishes two genera
RNA methyltransferase RsmE	BB_0062	1 aa	+	+	-	Distinguishes two genera
DNA mismatch repair protein mutL	BB_0211	4 aa	+	+	-	Distinguishes two genera
Hypothetical protein BRE314	BB_0227	1 aa	+	+	-	Distinguishes two genera
Methylgalactoside ABC transporter ATP-binding protein	BB_0318	1 aa	-	-	+	Distinguishes two genera
Sensory transduction histidine kinase	BB_0420	1 aa	-	-	+	Distinguishes two genera
Hypothetical protein Q7M860	BB_0455	2 aa	+	+	-	Distinguishes two genera
Hypothetical protein KK90081	BB_0083	1 aa	-	-	+	Distinguishes two genera
Outer membrane protein	BB_0167	1 aa	-	-	+	Distinguishes two genera
Transglycosylase SLT domain-containing protein	BB_0259	1 aa	-	-	+	Distinguishes two genera
Cell division protein FtsZ	BB_0299	1 aa	-	-	+	Distinguishes two genera
Excinuclease ABC subunit C	BB_0457	1 aa	-	-	+	Distinguishes two genera
Hypothetical protein BG0519	BB_0507	1 aa	-	-	+	Distinguishes two genera
Hypothetical protein BBIDN1270545	BB_0543	4 aa	-	-	+	Distinguishes two genera
Hypothetical protein BBUN400354	BB_0354	3 aa	-	-	+	Distinguishes two genera
Hypothetical protein BBUZS70553	BB_0543	1 aa	-	-	+	Distinguishes two genera
Hypothetical protein BB0554	BB_0554	1 aa	-	-	+	Distinguishes two genera
Hypothetical protein BB0554	BB_0554	2 aa	-	-	+	Distinguishes two genera
Hypothetical protein BBUCA803285	BB_0664	1 aa	-	-	+	Distinguishes two genera
Chemotaxis protein	BB_0681	1 aa	-	-	+	Distinguishes two genera
Hypothetical protein L14403475	BB_0707	1 aa	-	-	+	Distinguishes two genera
Membrane protein	BB_0234	1 aa	+	#	-	Insertion occurred after the branching of *B*. *turcica*
DNA polymerase III subunit -ta	BB_0455	2 aa	-	#	+	Insertion occurred after the branching of *B*. *turcica*
Hypothetical protein BB0838	BB_0838	3 aa	-	+	+	Deletion in the RF clade
Putative lipoprotein	BB_0227	3 aa	+	-	-	Insertion in the RF Clade
Hypothetical protein BRE355	BB_0353	1 aa	+	-	-	Insertion in the RF Clade
Hypothetical protein Q7M140	BB_0134	2 aa	+	-	-	Insertion in the RF Clade
Hypothetical protein BG0159	BB_0161	1 aa	-	+	+	Deletion in the RF clade
Methyl-accepting chemotaxis protein	BB_0681	2 aa	+	-	-	Insertion in the RF Clade
Chemotaxis protein	BB_0681	1 aa	-	+	+	Deletion in the RF clade

These CSIs were previously indicated to differentiate members of the genus *Borrelia* and *Borreliella* [6]. With the inclusion of sequence information for *B*. *turcica* and Candidadus Borrelia tachyglossi, these CSIs still differentiate the members of these two genera; however, some of them also show the deep branching of the RR group of species in comparison to the other *Borrelia* species. Abbreviations: RR–refer to the repitles- and echidna- related species *B*. *turcica* and Candidadus Borrelia tachyglossi RF–Relapsing Fever Clade; LD–Lyme Disease Clade; + = presence of insert;— = absence of insert

# Candidadus Borrelia tachyglossi contains the insert but it is absent in *B*. *turcica*.

**Fig 6:**
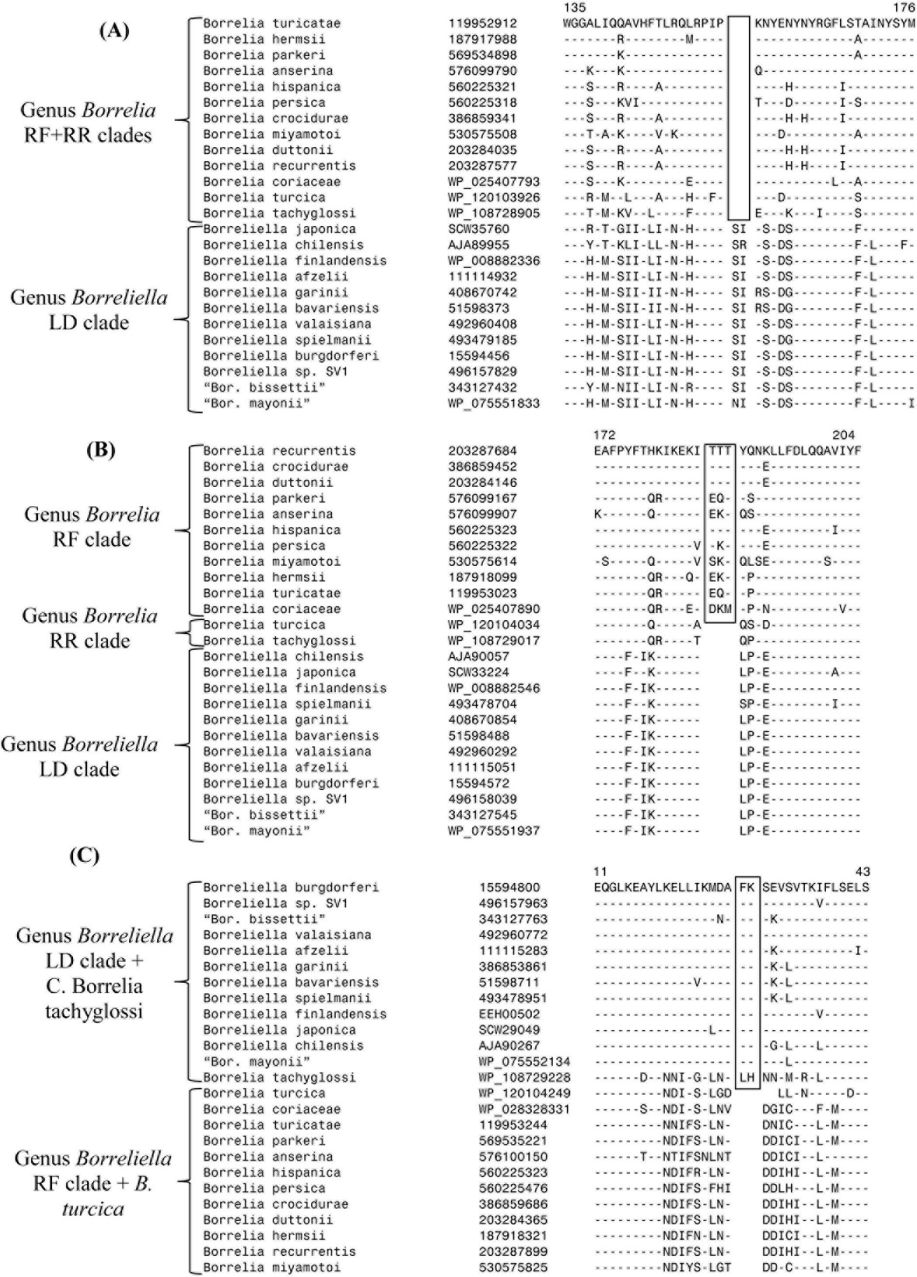
Partial sequence alignments of three CSIs in proteins found only in the *Borreliaceae* species providing differentiation among members of the genera *Borrelia* and *Borreliella*. (A) This panel shows a 2 aa CSI in a hypothetical protein BT0110 that differentiates the members of the genera *Borrelia* and *Borreliella*. Twenty nine other CSIs also show a similar species distribution (Table 3). Due to the absence of outgroup species it is difficult to infer whether this CSI is an insert in the genus *Borrelia* or a deletion in the genus *Borreliella*. (B) A 3 aa CSI in a putative lipoprotein that is specific for the RF clade of species. Due to the absence of this CSI in the LD clade as well as in *B*. *turcica* and Candidatus Borrelia tachyglossi homologs this CSI is an insert in the RF clade of species (see Fig 4). (C) A 2 aa CSI in DNA polymerase III subunit delta, which is commonly shared by the LD clade of species and Cand. Borrelia tachyglossi, but absent in *B*. *turcica* and the RF group of species. Based on its species distribution, this CSI is inferred to be an insert in a common ancestor of the RF clade and *B*. *turcica* (see Fig 4 for additional information).

If the genetic change leading to the CSI occurred in a common ancestor of either the LD group or the entire RF group (inclusive of the RR group) then the CSIs will be present in one of these groups and absent in the other, similar to that reported in the earlier work. Of the 38 CSIs in this category, 29 showed this pattern and they differentiate between the members of the two *Borreliaceae* genera. One example of a CSI of this kind is shown in Fig 6A. However, if the genetic change in a given gene/protein occurred in a common ancestor of the RF group after the divergence of the RR group of species (viz. *B*. *turcica* and Candidatus Borrelia tachyglossi), then such a CSI will be present in the RF group of species, but absent in *B*. *turcica* and Candidatus Borrelia tachyglossi as well as the LD group of species. There were 7 CSIs, which showed this type of pattern (listed at the bottom of Table 3). One example of a CSI showing this type of pattern is shown in Fig 6B. However, as indicated in Fig 4, the genetic changes in this CSI or other CSIs of this kind should not be interpreted as showing that *B*. *turcica* and Candidatus Borrelia tachyglossi are specifically related to the LD group of species, as these CSIs, due to the occurrence of genetic changes in a common ancestor of the RF group, are only distinguishing the RF group of species from other *Borreliaceae* species. Further, as noted earlier and shown in Fig 3, although in phylogenetic trees based on large datasets of proteins, *B*. *turcica* and Candidatus Borrelia tachyglossi form a deeper-branching clade, the grouping together of these two species/strain is not seen in trees based on several individual protein sequences and also in 16S rRNA trees (Fig 3B and 3C and unpublished results). Due to this, in some cases the genetic change leading to the CSI can also occur in an RF-group ancestor inclusive of *B*. *turcica* (or Candidatus Borrelia tachyglossi) but after the branching of Candidatus Borrelia tachyglossi (or *B*. *turcica*). The genetic changes in two of the CSIs in *Borreliaceae*-specific proteins (viz. a membrane protein and DNA polymerase III subunit delta) described in our earlier work [6] appeared to have occurred at these stages of evolution. Sequence information for one of these CSIs is presented in Fig 6C. In this case, the described CSI is present in LD clade of species and Cand. Borrelia tachyglossi whereas *B*. *turcica* and the RF group of species are lacking this CSI. However, in this case, it will again be incorrect to interpret that the presence of this CSI in Cand. Borrelia tachyglossi and the LD group of species indicates that this species is specifically related to the LD group of species. A summary of the distribution pattern of different CSIs in the second category before and after the inclusion of results from *B*. *turcica* and Cand. Borrelia tachyglossi is presented in Fig 4C and 4D. The CSIs whose specificities are questioned by Margos *et al*. [10] are marked by asterisk (*) in Fig 4. Based on the correct interpretation of the genetic and evolutionary significance of these CSIs, as shown in Fig 4, it is clear that these CSIs are also highly specific characteristics of most members of the genus *Borrelia*. In addition, they are also clarifying the phylogenetic placement of the species *B*. *turcica* and Cand. Borrelia tachyglossi within this genus and the family *Borreliaceae*.

Based on the evidence presented above, specifically, the correct interpretations of the results for the specificities of the CSIs and the inadequacy of genomic similarity (POCP threshold) as a criterion for genus level differentiation [12], it should be clear that the concerns raised by Margos *et al*. [10] to challenge the division of the genus *Borrelia* into two genera are not justified. In a recent publication, Estrada-Peña and Cabezas-Cruz [49] based on their examination of presence or absence of different biological processes in spirochetes species have inferred that members of the genus *Borrelia* and *Borreliella* are more similar to each other than other free-living (viz. *Sediminispirochaeta*, *Spirochaeta* and *Sphaerochaeta*) or pathogenic spirochetes such as *Leptospira*, *Treponema* and *Brachyspira*. However, their results are not surprising, as both *Borrelia* and *Borreliella* are part of the family *Borreliaceae* whose members exhibit very similar life cycle and vector(s)-host transmission characteristics [2,5,7,40]. With the exception of *B*. *recurrentis*, all other *Borrelieaceae* species have a tick-stage in their life cycle [2,5]. Thus, members of the genera *Borrelia* and *Borreliella* have coevolved intracellularly within their natural animal host-reservoir organisms for a long period of time. Due to this it is expected that all members of the family *Borreliaceae* (i.e. *Borrelia* and *Borreliella* genera) will share large number of biological processes and characteristics in common [5,47]. In our own work [6,7,41], we have described 31 CSIs and 82 CSPs which are uniquely shared by the members of these two genera. However, these shared characteristics are properties of the family and they reflect the multiple biological and phenotypic characteristics that the members of this family share in common. These shared properties and biological processes of the family *Borreliaceae*, which have been better studied, have likely led to the inference by Estrada-Peña and Cabezas-Cruz [49] that the members of these two genera are more closely related to each other than other spirochetes groups/genera. However, the observed similarity between these two genera, which are the shared properties of the family *Borreliaceae*, does not in any way minimizes or reduces the significance of large numbers of molecular, phenotypic and clinical differences that exist between the members of these two genera that are summarized in this work and which forms the basis of dividing this family into two different genera [6,7]. Estrada-Peña and Cabezas-Cruz [49] have not questioned the validity or significance of any these described characteristics and thus their resistance to splitting the family *Borreliaceae* is not justified.

To further clearly illustrate the differences between members of the genera *Borrelia* and *Borreliella*, in Table 4, I present a summary of some of the characteristics which distinguish members of these two genera. A number of other characteristics, which also distinguish these genera, are noted by Barbour [5] in a recent publication on the family *Borreliaceae*. The characteristics which distinguish members of these two genera include their different disease spectrums, multiple important differences in their epidemiology and phenotypic properties [5,7], and the clear differentiation and demarcation of these two groups based on genomic similarity and numerous molecular sequence based characteristics. Based on this evidence, it will be accurate to state that the distinction between these two groups of spirochetes is supported by more numerous and distinct types of characteristics than has been reported/observed for any two closely related groups (genera) of prokaryotes. Hence, we urge critics of this division to keep in mind the strong and incontrovertible evidence supporting the distinctness of these two groups of spirochetes.

**Table 4:** Clinical, molecular and phenotypic differences between members of the RF-RR group (genus *Borrelia*) and the Lyme disease group (genus *Borreliella*).

Characteristics	Genus *Borrelia* (RF+RR species)	Genus *Borreliella* (LD group)	References
***Clinical Spectrum***			
Relapsing fever causing bacteria	Encompasses All	None	See [1,2,5,50,51]
Lyme-disease causing bacteria	None	Encompasses All	See [1,2,5,50,52]
***Phylogenetic*, *Molecular and Genomic Characteristics***			
Branching in phylogenetic trees based on 16S rRNA and other genes/proteins sequences	In all phylogenetic trees, members of the RF+RR group and the LD group form strongly-supported clades clearly separated from each other.		[5–7,10,53] present study
Average Nucleotide Identity (ANI) Matrix based on Genomes	Members of the RF+RR group and the LD group are clearly differentiated based on higher similarity seen between the members of each group.		[6]
Average Amino Acid Identity (AAI) Matrix based on Genomes	Members of the RF+RR group and the LD group are clearly differentiated based on higher similarity seen between the members of each group.		[7]
Percentage of Conserved Protein (POCP) Matrix based on Genome Sequences	Members of the RF+RR group and the LD group are clearly differentiated based on higher similarity seen between the members of each group.		Present study
Conserved Signature Indels (CSIs) in widely-distributed proteins	4 CSIs exclusively found in different members of this group. 4 other CSIs are also specific for the RF clade but lacking in deeper branching RR group of species.	7 CSIs exclusively found in the LD group differentiating it from the RF+RR group of species.	[6,7] and present study
Conserved Signature Indels (CSIs) in Borreliaceae-specific proteins	29 CSIs provide clear differentiation between these two groups of species + 9 CSIs specific for the RF clade and show deeper branching of the RR group of species.		Present study
Conserved Signature Proteins	4 CSPs exclusively found in most members of this group.	17 CSPs exclusively (or mainly) found in members of this group.	[6]^#^
***Phenotypic Characteristics***			
Arthropod vectors	Argasid ticks, prostriate and metastriate ixodid ticks and human body louse*	Primarily prostriate ticks of the genus *Ixodes*	See [1,2,5,50–52,54]
Density of Spirochetes in blood of infected humans/animals	High	Low	See [5,7,51,52]
Average number of flagella at one end of cells	Mostly in the range of 15–20	Generally in the range of 7–11	See [5,7,51,52]

In addition to the characteristics noted in this Table, some other molecular and phenotypic differences between members of these two genera have been summarized by Barbour [5].

^#^ Based on updated sequence information.

* only *B*. *recurrentis* is transmitted via a louse.

As noted earlier, the species which are now part of the genus *Borreliella* are widely referred to by the name "*Borrelia burgdorferi sensu lato*", recognizing their distinctness from other *Borreliaceae* species, which are members of the genus *Borrelia* [1,2,5,9]. However, the meanings of the terms "*Borrelia burgdorferi sensu lato*" or “RF clade”, or which *Borrelia* species are part of each of these groups, or the species which fall outside of these two groups (viz. RR group of species), are not clearly understood by many scientists and others professionals working in this as well as other related fields. Hence, the substitution of these poorly understood terms with more precise and unambiguous names (*Borrelia* and *Borreliella*), which clearly differentiates the relapsing fever encompassing group of species from the different Lyme disease-causing and related microorganisms [3,4,46], should be highly beneficial to the field in terms of advancing our understanding of the molecular, biochemical and biological differences that underlie these two unique disease-causing groups of microorganisms.

Subsequent to our earlier work [6], a number of new species belonging to the family *Borreliaceae* have been described [55–58]. Of these species, *Borrelia bissettiae*, *Borrelia californiensis*, *Borrelia lanei*, *Borrelia mayonii* and *Borrelia yangtzensis* group reliably with the members of the genus *Borreliella* (LD-group) in 16S rRNA trees [55] (Fig 3C), or where genome sequence information is available based on uniquely shared molecular characteristics with other members of the genus *Borreliella* [8,9] (see Table 1). Hence, new name combinations for these species are described below.

**Description of *Borreliella bissettiae* comb. nov.** (bis.set´ti.ae. N.L. gen. n. *bissettiae*, of Bissett, named after Marjorie L. Bissett, who isolated and described this spirochaete with her co-worker Warren Hill) Basonym: *Borrelia bissettiae* Margos et al. 2016

The strain for this species was isolated by Bissett and Hill [59] and the description of this species is as provided by Margos *et al*. [56] for *Borrelia bissettiae*

Type strain: DN127 = DSM 17990 = CIP 109136.

**Description of *Borreliella californiensis* comb. nov. (**ca.li.for.ni.en´sis. N.L. fem. adj. *californiensis*, belonging to California, from where the type strain was isolated)

Basonym: *Borrelia californiensis* Margos et al. 2016

The description of this species is the same as provided by Postic *et al*. [60] and Margos *et al*. [56] for *Borrelia californiensis*

Type strain: CA446 = DSM 17989 = ATCC BAA-2689.

**Description of *Borreliella lanei* comb. nov.** (la.ne′i. N.L. gen. n. *lanei*, in honour of Professor Robert S. Lane for his outstanding contributions to *Borrelia* and *Ixodes* research)

Basonym: *Borrelia lanei* Margos et al. 2017

The description of this species is the same as provided by Margos *et al*. [55] for *Borrelia lanei*

Type strain: (see also StrainInfo.net) CA28-91 = DSM 17992 = CIP 109135.

**Description of *Borreliella mayonii* comb. nov.** (ma.yo′ni.i. N.L. gen. n. *mayonii*, after William James Mayo and Charles Horace Mayo, founders of the Mayo Clinic).

Basonym: *Borrelia mayonii* Pritt et al. 2016

The description of this species is the same as provided by Pritt *et al*. [58] for *Borrelia mayonii*

Type strain: MN14-1420 = ATCC BAA-2743 = DSM 10281.

**Description of *Borreliella yangtzensis* comb. nov.** (yang.tzen′sis. N.L. fem. adj. *yangtzensis*, referring to the Yangtze River valley in China, where these organisms were first isolated.

Basonym: *Borrelia yangtzensis* Margos et al. 2015

The description of this species is the same as provided by Margos *et al*. [57] for *Borrelia yangtzensis*

Type strain: Okinawa-CW62 = DSM 24625 = JCM 17189.

## Supporting information

S1 Table Species and genome sequence information for *Enterobacteriaceae* species used in POCP analysis. (PDF)

S2 Table Species and genome sequence information for *Morganellaceae* and *Cystobacteraceae* species used in POCP analysis. (PDF)

S3 Table Averages of the intrageneric and intergeneric POCP values for species from the family *Morganellaceae* and *Cystobacteraceae*. (PDF)
