# Supplementary material for: Distinction between Borrelia and Borreliella is more robustly supported by molecular and phenotypic characteristics than all other neighbouring prokaryotic genera: Response to Margos' et al. "The genus Borrelia reloaded" (PLoS ONE 13(12): e0208432)
Source: PLoS One. 2019 Aug 27;14(8):e0221397. doi: 10.1371/journal.pone.0221397 (PMC6711536; doi:10.1371/journal.pone.0221397)
Supplement: S1 Table — (PDF) [file pone.0221397.s001.pdf]

S1 Table

Species and genome sequence information for *Enterobacteriaceae* species used in POCP analysis.

Organism	Type Strain?	GenBank Accession	Size (Mb)
Atlantibacter hermannii NBRC 105704	Y	GCA_000248015.2	4.50
Buttiauxella agrestis ATCC 33320	Y	GCA_000735355.1	4.73
Buttiauxella brennerae ATCC 51605	Y	GCA_001654925.1	4.75
Buttiauxella ferragutiae ATCC 51602	Y	GCA_001654915.1	5.09
Buttiauxella gaviniae ATCC 51604	Y	GCA_001654835.1	5.07
Buttiauxella noackiae ATCC 51607	Y	GCA_001654865.1	4.80
Cedecea davisae DSM 4568	Y	GCA_000412335.2	4.89
Cedecea neteri ATCC 33855	Y	GCA_001571265.1	5.20
Citrobacter amalonaticus Y19	N	GCA_000981805.1	5.88
Citrobacter braakii GTA-CB04	N	GCA_000786275.1	5.04
Citrobacter farmeri GTC 1319	N	GCA_000764735.1	4.93
Citrobacter freundii ATCC 8090	Y	GCA_000312465.1	5.00
Citrobacter koseri ATCC BAA-895	N	GCA_000018045.1	4.74
Citrobacter pasteurii CIP 55.13	Y	GCA_000826205.1	4.99
Citrobacter rodentium ATCC 51459	Y	GCA_000835925.1	5.39
Citrobacter sedlakii NBRC 105722	N	GCA_000759835.1	4.63
Citrobacter werkmanii NBRC 105721	N	GCA_000759755.1	4.95
Citrobacter youngae ATCC 29220	N	GCA_000155975.1	5.15
Cronobacter condimenti 1330	Y	GCA_001277255.1	4.50
Cronobacter dublinensis LMG 23823	Y	GCA_001277235.1	4.63
Cronobacter malonaticus LMG 23826	Y	GCA_001277215.2	4.47
Cronobacter muytjensii ATCC 51329	Y	GCA_001277195.1	4.36
Cronobacter sakazakii ATCC 29544	Y	GCA_000982825.1	4.66
Cronobacter universalis NCTC 9529	Y	GCA_001277175.1	4.44
Enterobacter aerogenes KCTC 2190	Y	GCA_000215745.1	5.28
Enterobacter asburiae ATCC 35953	Y	GCA_001521715.1	4.81
Enterobacter cancerogenus ATCC 35316	N	GCA_000155995.1	4.64
Enterobacter cloacae ATCC 13047	Y	GCA_000025565.1	5.60
Enterobacter hormaechei ATCC 49162	Y	GCA_000213995.1	4.86
Enterobacter kobei DSM 13645	Y	GCF_001729765.1	4.93
Enterobacter lignolyticus SCF1	Y	GCF_000164865.1	4.81
Enterobacter ludwigii EN-119	Y	GCA_000818595.1	4.95
Enterobacter massiliensis JC163	Y	GCA_000321045.2	5.00
Enterobacter mori LMG 25706	Y	GCA_000211415.1	4.96
Enterobacter soli ATCC BAA-2102	Y	GCF_001654845.1	4.96077
Enterobacter xiangfangensis LMG27195	Y	GCF_001729785.1	4.66
Escherichia albertii KF1	N	GCA_000512125.1	4.70
Escherichia coli O157:H7 str. Sakai	N	GCA_000008865.1	5.59
Escherichia coli str. K-12 substr. MG1655	N	GCA_000005845.2	4.64
Escherichia fergusonii ATCC 35469	Y	GCA_000026225.1	4.64
Escherichia marmotae HT073016	Y	GCA_000807695.3	4.51
Escherichia vulneris NBRC 102420	Y	GCA_000759795.1	4.37

Franconibacter helveticus LMG 23732	Y	GCA_000463115.2	4.52
Franconibacter pulveris DSM 19144	Y	GCA_000621185.1	4.71
Klebsiella michiganensis E718	N	GCA_000276705.2	6.56
Klebsiella michiganensis H1g *	N	GCA_000633235.1	5.84
Klebsiella michiganensis HKOPL1 *	N	GCA_000632415.1	5.91
Klebsiella michiganensis KCTC 1686 *	N	GCA_000240325.1	5.97
Klebsiella michiganensis M1 *	N	GCA_000724525.1	6.31
Klebsiella michiganensis M5al *	N	GCA_001633115.1	5.78
Klebsiella michiganensis RC10 *	N	GCA_000963575.1	5.11
Klebsiella oxytoca KCTC 1686	N	GCA_000240325.1	5.97
Klebsiella pneumoniae ATCC 13883	Y	GCA_000788015.1	5.73
Klebsiella quasipneumoniae 01A030	Y	GCA_000751755.1	5.47
Klebsiella variicola DSM 15968	Y	GCF_000828055.2	5.52
Kluyvera ascorbata ATCC 33433	Y	GCA_000735365.1	4.93
Kluyvera cryocrescens NBRC 102467	Y	GCA_001571285.1	5.04
Kluyvera georgiana ATCC 51603	Y	GCA_001654985.1	5.07
Kluyvera intermedia CAV1151 *	N	GCF_001022135.1	6.17
Kluyvera intermedia NBRC 102594	Y	GCA_001598315.1	4.64
Kluyvera intestini GT-16	Y	GCA_001856865.1	5.87
Kosakonia cowanii JCM 10956	Y	GCA_001312885.1	4.81
Kosakonia oryzae Ola 51	Y	GCF_001658025.1	5.30
Kosakonia radicincitans DSM 16656	Y	GCA_000280495.1	6.06
Kosakonia sacchari SP1	Y	GCA_000300455.4	4.90
Leclercia adecarboxylata ATCC 23216	Y	GCA_000735515.1	4.99
Lelliottia amnigena NBRC 105700	Y	GCA_001514515.1	4.43
Lelliottia nimipressuralis CIP 104980	Y	GCA_001875645.1	4.98
Mangrovibacter sp. MFB070	N	GCA_000705335.1	5.36
Pluralibacter gergoviae ATCC 33028	Y	GCA_001598855.1	5.66
Raoultella ornithinolytica NBRC 105727	N	GCA_001598295.1	5.53
Raoultella planticola ATCC 33531	Y	GCA_000735435.1	5.67
Salmonella bongori NCTC 12419	Y	GCA_000252995.1	4.46
Salmonella enterica LT2	Y	GCA_000006945.2	4.95
Shigella boydii Sb227	N	GCA_000012025.1	4.65
Shigella dysenteriae Sd197	N	GCA_000012005.1	4.56
Shigella flexneri 301	N	GCA_000006925.2	4.83
Shigella sonnei 53G	N	GCA_000283715.1	5.22
Shigella sonnei Ss046	N	GCA_000092525.1	5.06
Shimwellia blattae DSM 4481	Y	GCA_000262305.1	4.16
Siccibacter colletis 1383	Y	GCA_000696575.1	4.26
Siccibacter turicensis LMG 23730	Y	GCA_000463155.2	4.24
Trabulsiella guamensis ATCC 49490	Y	GCA_000734965.1	4.93
Trabulsiella odontotermitis TbO2.3	N	GCA_001297765.1	4.60
Yokenella regensburgei ATCC 49455	Y	GCA_000735455.1	4.85
Dickeya zeae NCPPB 2538	Y	GCA_000406165.1	4.56
Arsenophonus nasoniae DSM 15247	Y	GCA_000429565.1	3.67
Hafnia alvei ATCC 13337	Y	GCA_000735375.1	4.82
