# Supplementary material for: Distinction between Borrelia and Borreliella is more robustly supported by molecular and phenotypic characteristics than all other neighbouring prokaryotic genera: Response to Margos' et al. "The genus Borrelia reloaded" (PLoS ONE 13(12): e0208432)
Source: PLoS One. 2019 Aug 27;14(8):e0221397. doi: 10.1371/journal.pone.0221397 (PMC6711536; doi:10.1371/journal.pone.0221397)
Supplement: S2 Table — (PDF) [file pone.0221397.s002.pdf]

S2 Table

Species and genome sequence information for *Morganellaceae* and *Cystobacteraceae* species used in POCP analysis.

Species Name	**Accession Number**
Xenorhabdus cabanillasii JM26	GCA_000531755.1
Xenorhabdus hominickii ANU1	GCA_001721185.1
Xenorhabdus szentirmaii DSM 16338	GCA_000531455.1
Xenorhabdus nematophila ATCC 19061	GCA_000252955.1
Xenorhabdus poinarii strain G6	GCA_000968175.1
Xenorhabdus doucetiae FRM16	GCA_000968195.1
Xenorhabdus griffiniae BMMCB	GCA_001028135.1
Xenorhabdus thuongxuanensis 30TX1	GCA_001908095.1
Xenorhabdus eapokensis DL20	GCA_001908105.1
Xenorhabdus khoisanae MCB	GCA_001037465.1
Xenorhabdus bovienii SS-2004	GCA_000027225.1
Photorhabdus heterorhabditis VMG	GCA_001280945.1
Photorhabdus asymbiotica ATCC 43949	GCA_000196475.1
Photorhabdus temperata subsp. thracensis	GCA_001010285.1
Photorhabdus luminescens ATCC 29999	GCA_000931955.2
Arsenophonus nasoniae DSM 15247	GCA_000429565.1
Moellerella wisconsensis ATCC 35017	GCA_001294465.1
Providencia alcalifaciens DSM 30120	GCA_000173415.1

Providencia rustigianii DSM 4541	GCA_000156395.1
Providencia heimbachae ATCC 35613	GCA_001655055.1
Providencia rettgeri RB151	GCA_001874625.1
Providencia sneebia DSM 19967	GCA_000314895.2
Providencia burhodogranariea DSM 19968	GCA_000314855.2
Providencia stuartii MRSN 2154	GCA_000259175.1
Morganella morganii subsp. morganii NBRC 3848	GCA_001598895.1
Cosenzaea myxofaciens ATCC 19692	GCA_001654855.1
Proteus mirabilis ATCC 29906	GCA_000160755.1
Proteus hauseri ATCC 700826	GCA_001654965.1
Proteus penneri ATCC 35198	GCA_000155835.1
Proteus vulgaris ATCC 49132	GCA_000754995.1

Species Name	Accession Number
Anaeromyxobacter dehalogenans	GCA_000022145.1
Vulgatibacter incomptus	GCA_001263175.1
Archangium gephyra	GCA_001027285.1
Archangium violaceum Cb vi76	GCA_000733295.1
Melittangium boletus DSM 14713	GCA_002305855.1

Cystobacter fuscus	GCA_000335475.2
Cystobacter violaceus	GCA_000733295.1
Hyalangium minutum DSM 14724	GCA_000737315.1
Stigmatella erecta DSM 16858	GCA_900111745.1
Stigmatella aurantiaca	GCA_000165485.1
Corallococcus coralloides DSM 2259	GCA_000255295.1
Myxococcus stipitatus	GCA_000331735.1
Myxococcus hansupus	GCA_000280925.3
Myxococcus xanthus	GCA_000012685.1
Myxococcus virescens DSM 2260	GCA_900101905.1
Myxococcus fulvus	GCA_000988565.1
Myxococcus macrosporus DSM 14697	GCA_002305895.1
Sandaracinus amylolyticus	GCA_000737325.2
Minicystis rosea DSM 24000	GCA_001931535.1
Sorangium cellulosum	GCA_000067165.1
Chondromyces crocatus	GCA_001189295.1
Chondromyces apiculatus	GCA_000601485.1
Labilithrix luteola	GCA_001263205.1
Pajaroellobacter abortibovis BTF92-0548A 99-	GCA_001931505.1

0131	
Haliangium ochraceum	GCA_000024805.1
Nannocystis exedens ATCC 25963	GCA_002343915.1
Enhygromyxa salina DSM 15201	GCA_000737335.3
Plesiocystis pacifica	GCA_000170895.1
