# Supplementary material for: Distinction between Borrelia and Borreliella is more robustly supported by molecular and phenotypic characteristics than all other neighbouring prokaryotic genera: Response to Margos' et al. "The genus Borrelia reloaded" (PLoS ONE 13(12): e0208432)
Source: PLoS One. 2019 Aug 27;14(8):e0221397. doi: 10.1371/journal.pone.0221397 (PMC6711536; doi:10.1371/journal.pone.0221397)
Supplement: S3 Table — (PDF) [file pone.0221397.s003.pdf]

S3 Table

Average interspecies and intergeneric POCP values for different genome sequenced species from the family *Morganellaceae* and *Cystobacteraceae*

(A) Average POCP Matrix - Family *Morganellaceae*

Genera	*Xenorhabdus*	*Photorhabdus*	*Arsenophonus*	*Moellerella*	*Providencia*	*Morganella*	*Cosenzaea*	*Proteus*
Xenorhabdus	0.794	0.698	0.599	0.637	0.609	0.617	0.625	0.616
Photorhabdus	0.698	0.865	0.698	0.587	0.625	0.598	0.603	0.598
Arsenophonus	0.599	0.698	n/a	0.671	0.607	0.620	0.639	0.609
Moellerella	0.637	0.587	0.671	n/a	0.763	0.708	0.746	0.717
Providencia	0.609	0.625	0.607	0.763	0.806	0.707	0.716	0.713
Morganella	0.617	0.598	0.620	0.708	0.707	n/a	0.738	0.739
Cosenzaea	0.625	0.603	0.639	0.746	0.716	0.738	n/a	0.845
Proteus	0.616	0.598	0.609	0.717	0.713	0.739	0.845	0.873

(B) Average POCP Matrix - Family *Cystobacteraceae*

	Archangium	*Melittangium*	*Cystobacter*	*Hyalangium*	*Stigmatella*	*Corallococcus*	*Myxococcus*
Archangium	0.8521	0.7300	0.8243	0.6892	0.6370	0.6473	0.6288
Melittangium	0.7300	n/a	0.7654	0.6959	0.7047	0.6765	0.6406
Cystobacter	0.8243	0.7654	0.7099	0.6744	0.6543	0.6488	0.6187
Hyalangium	0.6892	0.6959	0.6744	n/a	0.7699	0.7007	0.6800
Stigmatella	0.6370	0.7047	0.6543	0.7699	0.8946	0.7018	0.6699
Corallococcus	0.6473	0.6765	0.6488	0.7007	0.7018	n/a	0.7552
Myxococcus	0.6288	0.6406	0.6187	0.6800	0.6699	0.7552	0.8658

The shaded values along the diagonal shows the average POCP values for different species within a given genus (i.e. interspecies values), whereas all other values represent average intergeneric POCP values for different genera within these families. n/a indicates that the interspecies values were not calculated as a single species was present in these cases.
